# Contactless Credit Cards Payment Fraud Protection by Ambient Authentication

**DOI:** 10.3390/s22051989

**Published:** 2022-03-03

**Authors:** Ming-Hour Yang, Jia-Ning Luo, Murugesan Vijayalakshmi, Selvaraj Mercy Shalinie

**Affiliations:** 1 Department of Information and Computer Engineering, Chung Yuan Christian University, Taoyuan 32023, Taiwan; 2 Department of Computer Science and Information Engineering, Chung Cheng Institute of Technology, National Defense University, Taoyuan 335009, Taiwan; deer@ccit.ndu.edu.tw; 3 Department of Computer Science and Engineering, Thiagarajar College of Engineering, Madurai 625015, India; mviji@tce.edu (M.V.); shalinie@tce.edu (S.M.S.)

**Keywords:** NFC, smart card, IoT security, mutual authentication, mobile transaction, ambient authentication

## Abstract

In recent years, improvements to the computational ability of mobile phones and support for near-field-communication have enabled transactions to be performed by using mobile phones to emulate a credit card or by using quick response codes. Thus, users need not carry credit cards but can simply use their mobile phones. However, the Europay MasterCard Visa (EMV) protocol is associated with a number of security concerns. In contactless transactions, attackers can make purchases by launching a relay attack from a distance. To protect message transmission and prevent relay attacks, we propose a transaction protocol that is compatible with EMV protocols and that can perform mutual authentication and ambient authentication on near-field-communication-enabled mobile phones. Through mutual authentication, our protocol ensures the legitimacy of transactions and establishes keys for a transaction to protect the subsequent messages, thereby avoiding security problems in EMV protocols, such as man-in-the-middle attacks, skimming, and clone attacks on credit cards. By using ambient factors, our protocol verifies whether both transacting parties are located in the same environment, and it prevents relay attacks in the transaction process.

## 1. Introduction

Mobile payment can be classified into three types according to the technology it uses [1]. The first type entails the short message service (SMS); transactions can be completed simply by sending an SMS without the use of a credit card or a bank account. However, transactions performed using SMS payments are therefore vulnerable to eavesdropping or tampering [2]. For this reason, SMS is rarely offered for transactions except, occasionally, for making donations to charity organizations. The second type of mobile payment involves a quick response code (QR code). Nonetheless, the susceptibility of QR code payment to structured query language injection, command injection, and phishing [3], coupled with the convenience of shopping, has engendered consumer acceptance of transactions involving near-field-communication (NFC) technology [1]. The third type is NFC-based mobile payment. Mobile phones adopt NFC card-emulation mode to emulate a secure transaction environment for Europay MasterCard Visa (EMV) contactless chip cards, thus enabling conventional credit cards to be stored in NFC-based phones as virtual cards for mobile payment. To strengthen transaction security by preventing the direct interception of credit card numbers and to protect user privacy, EMVCo [4] proposed using token numbers in place of primary account numbers (PANs), which are fixed credit card numbers. Token numbers change for every new transaction process to avoid replay attacks, in which attackers intercept another person’s PAN from a single transaction and use this PAN to perform transactions with other merchants [5].

A number of security problems continue to affect EMV contactless card payment standards [6]. Attackers can adopt man-in-the-middle (MITM) attacks to impersonate EMV credit cards in response to the authentication approval message of a merchant’s point of sale (POS) terminal. Attackers then enter an arbitrary personal identification number (PIN) to complete a transaction, rendering the PIN of the original credit card invalid [7,8]. Because EMV has not mandated the encryption of credit card transaction messages, attackers can steal credit card numbers and other personal information [9,10] and, in turn, access transaction information that facilitates attackers’ online [9] and offline [11] use of a credit card stolen from the original cardholder. If a credit card uses dynamic data authentication (DDA) or combined DDA/application cryptogram generation (CDA) to protect and confirm the source of transaction messages, then the data freshness of every transaction can be ensured to avoid replay attacks because the Dynamic Data Authentication Data Object List (DDOL), which is a transaction message generated through DDA regulation, must contain Signed Dynamic Application Data (SDAD), which is composed of POS-generated random numbers, DDOL-designated POS dynamic data, and dynamic numbers generated with time-variant parameters. However, because the subsequent transaction message is unsigned, verifying that the subsequent transaction is conducted by the same card remains impossible [12]. For CDA, because transaction messages are not included in a card reader’s identification information, malicious attackers can impersonate an automated teller machine (ATM) or POS and send a message regarding the expected time of attack to trick a user’s card into performing transactions. Moreover, random number generators used in POS and ATMs can predict the subsequent random number; this type of attack is called a preplay attack [13,14,15]. To address this problem, Yang developed an EMV credit card protocol (EPMAR) [16], using mutual authentication to verify the POS identity of the store communicating with the card. This protocol can prevent attackers from masquerading as a POS to preplay messages. With a session key, the protocol avoids the problem whereby the same random number can generate identical messages regarding legitimate transactions. Tso’s protocol further enhanced consumer privacy [17].

In this study, we propose a transaction protocol that is applicable to devices, compatible with EMV protocols, requires no alteration of user behavior, and avoids relay attacks on ambient authentication. Moreover, the proposed protocol can address the following security problems:

1. Cardholder authentication problems: Murdoch et al. [8] indicated that, in EMV protocols, because a card reader and credit card are not synchronized for PIN verification and signature verification, attackers are able to enter arbitrary PINs to complete a transaction.
2. EMV authenticates only the card, not the POS terminal: Liu et al. [7] and Murdoch et al. [8] indicated that attackers can access card information by using EMV-supported readers.
3. Transaction message is not encrypted: Haselsteiner et al. [9] and Hancke et al. [10] identified that an unencrypted transaction message communicated through NFC is susceptible to skimming. Attackers can use this message to successfully perform online [9] or offline [11] transactions.
4. Preplay attacks: Attackers use prerecorded transaction messages to perform transactions with the same random number and payment amount [13,14,15].
5. Relay attacks: Attackers holding a device in close proximity to a POS terminal forward a transaction to the device performing the real transaction from a distance, and the merchant unknowingly completes the transaction with a remote victim’s credit card.

Section 2 of this paper discusses the attack model, Section 3 summarizes the related works, Section 4 of this paper presents a discussion of our proposed method, Section 5 describes our ambient authentication experimental results, Section 6 provides a security analysis of our proposed protocol, and Section 7 concludes this study and provides future research directions.

## 2. Attack Model

**Figure 1:**
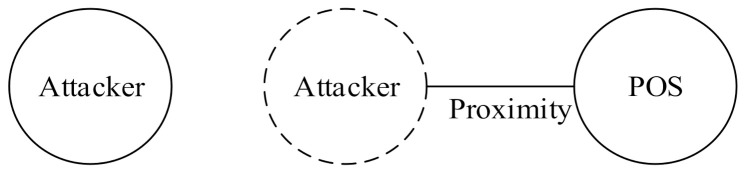
Distance fraud attack.

Attackers can, nonetheless, launch a relay attack, in which an attacker holding a device near a POS terminal forwards a transaction to the device performing the actual transaction from a distance, and the merchant unknowingly completes a transaction with a remote victim’s credit card. Relay attacks can be divided into three types according to the degree of influence and impact [18]. The first type of relay attack is the distance fraud attack [19] or wormhole attack [20], as illustrated in Figure 1; an attacker’s transaction device shortens message transmission time by relaying a POS demand in advance. This enables the attacker to misrepresent the distance between a POS and a transaction device as being shorter than it is. This type of attack is realistically unlikely to result in monetary loss because merchants are usually aware that the consumer is physically absent. 

**Figure 2:**
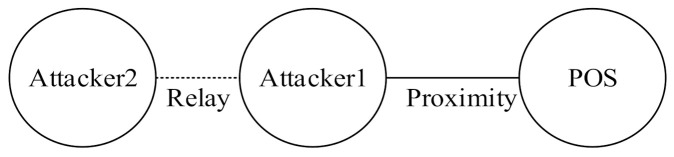
Terrorist fraud attack.

The second type of relay attack is the terrorist fraud attack [21], as shown in Figure 2. Attacker 1 relays a transaction message to Attacker 2, who is farther away, leading the merchant’s POS to believe that Attacker 1 is in close proximity, and the merchant therefore completes the transaction with Attacker 1. In this attack mode, Attacker 2, who completes the transaction, is proof that he or she (Attacker 2) is not at the purchase location, and the bank may thus have to bear the cost of consumption. However, the impact of this attack is relatively limited because the attacker must partake in all dispute-handling processes, increasing his or her identity exposure. 

**Figure 3:**
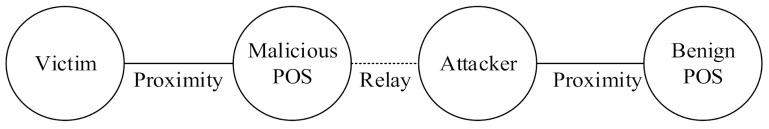
Mafia fraud attack.

The third type of relay attack is a mafia fraud attack, which has the largest impact. As shown in Figure 3, an attacker initiates an authentication request by using the Malicious POS controlled by the attacker and a victim’s credit card. The attacker then sends information that he or she obtains to the device of another attacker who is farther away. This type of attack was first developed by Desmedt et al. [22]. 

## 3. Related Works

As described in the previous attack model, Mafia fraud attacks have been proven by numerous studies to successfully facilitate the unauthorized use of a victim’s credit card for contactless payments [23,24,25] or NFC-based phones [5,26,27,28,29,30,31]. Because credit cards in general are not equipped with a block-reading function, attackers can complete numerous contactless transactions with a victim’s credit card in a crowded environment, such as the Taipei Metro, during rush hour. Frequent launches of this type of attack pose problems for financial order.

To solve relay attack problems, Chothia et al. developed a PaySafe protocol to ensure that a merchant’s POS and the credit card with which the transaction is performed are in close proximity [32]. Thereafter, in 2015, EMV integrated distance-bounding protocols into contactless card transaction standards to detect relay attacks [33]. However, card response time and card transaction time must be a maximum of 0.1 s and 0.5 s, respectively [34]; therefore, a highly accurate timer and special hardware are required to calculate message response time to detect relay attacks and avoid false positive and false negative results [35]. Studies have focused on the use of built-in mobile-phone sensors to verify whether a consumer’s payment device is located in the same environment as a merchant’s POS. 

Ambient authentication involves, for example, the use of global positioning systems (GPSs), Bluetooth, Wi-Fi, audio sensors, light, or temperature sensors [35,36,37,38,39,40] in mobile phones to verify that two devices are at the same location and thus reduce the threat of relay attacks. Mehrnezha et al. compared the similarity of the accelerometer data measured when the mobile phone is used to tap the POS to ensure that both devices are at the same location [39]. However, this solution is only effective if users change the manner in which they use credit cards. Halevi et al. proposed a detection method that uses sound or light [35]; when sampling ambient factors, Halevi et al. did not account for the sound randomly generated by the POS terminal at the time or the sound used in combination with an ambient-light-sensing message. For this reason, attackers can forward a transaction message to other locations with similar ambient audio and light factors to pass authentication, or attackers can relay previously recorded ambient authentication information to bypass authentication checks. Ma et al. proposed the use of GPS to verify that a POS and credit card-linked phone are at the same location [37]. For this solution, Ma et al. did not indicate methods to integrate sensing information into EMV protocols and achieve compatibility with existing EMV protocols, nor did they prove their method to be resistant to the majority of currently known attacks on online and offline card transactions.

## 4. An Ambient Authentication Payment Fraud Protection Method Compatible with EMV Card Protocol

We propose an EMV-compatible transaction protocol that avoids the distance fraud attack, the terrorist attack, and the Mafia fraud attack. We used only current EMV commands and adopted the reserved-for-future-use (RFU) field in the command parameter to send the message we added, thereby further securing user–merchant transactions. The proposed protocol performs a transaction through the EMV transaction protocol and is integrated with ambient authentication to avoid relay attacks.

The EMPAS protocol involves the same participating roles as the original EMV protocol. These include the acquiring bank, issuing bank, token service provider (TSP), user with NFC-enabled phone, and merchant (POS). Assumptions for their functions and environment are defined as follows:

1. User’s NFC-enabled phone: This has a secure storage environment to ensure that it possesses an SE or HCE-sensitive data (including tokens, keys, and programs deployed within).
2. TSP: In the initial phase, the TSP generates a unique token number, and the token is securely sent to a secure storage environment in the user’s phone.
3. Acquiring bank: This bank is responsible for deploying a POS to merchants. It connects to the issuing bank through a financial network to verify the transaction information.
4. Issuing bank: This cooperates with the TSP and is responsible for managing user’s cards.
5. Merchant: This uses an acquiring bank’s POS terminal and user’s NFC-enabled phone to send transaction information to the acquiring bank through a financial network.

The symbols and their definitions are presented in Table 1.

**Figure 4:**
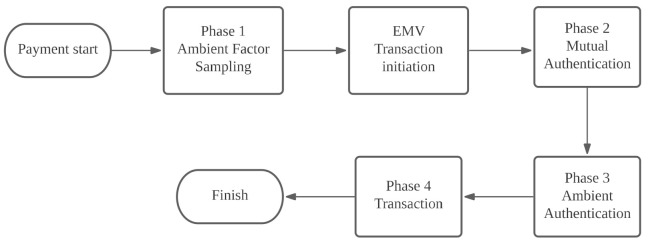
EMPAS transaction process.

Figure 4 illustrates the four transaction phases when users use mobile devices to pay with their credit card. Phase 1 is the ambient factor sampling phase. Before a phone and POS commence the EMV protocol, accelerometer values change when users tap their phones to the POS terminal, and the POS terminal requests audio sampling by the user and merchant. This phase occurs before the EMV transaction protocol is initiated; it therefore does not include the sampling time as adopted for EMV’s maximum 500 ms transaction time requirement. Phase 2 is the mutual authentication phase, in which the user and merchant use their certificate to authenticate each other. Phase 3 is the ambient authentication phase: After the merchant receives the user’s ambient samples, the ambient samples of both parties are compared to verify that both transacting parties are at the same location. The actual transaction phase ensues when authentication is successful; otherwise, transaction is terminated. Phase 4 is the transaction phase.

Phase 1 Ambient Factor Sampling Phase

Consumers swipe their credit cards by tapping their mobile phones to the merchant’s POS terminal, changing the POS terminal’s accelerometer value. At this point, the POS terminal randomly generates a soundwave of frequency 18 to 22 KHz that is imperceptible to the human ear but supported by general phone speakers. The terminal then sends out a message requesting that the phone conduct sound sampling. After the message is sent, the POS terminal plays this soundwave as a challenge message. After a consumer’s phone receives the challenge, the phone and POS concurrently record the ambient sound for 1 s. At the end of sampling, the phone stores the audio samples and valid time in a secure storage environment. Thus, for every transaction, this approach can prevent attackers from forwarding transaction messages to a place with a similar environment and from replaying previously recorded ambient sounds to pass authentication.

Phase 2 Mutual Authentication Phase

**Figure 5:**
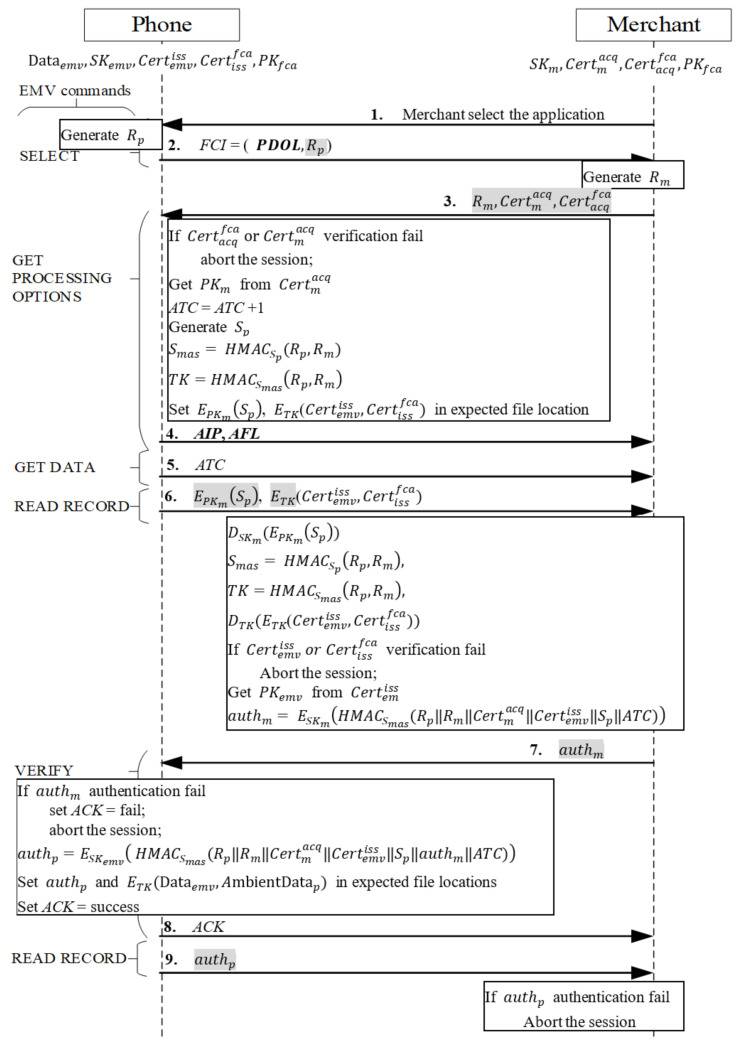
Mutual authentication phase.

When users initiate a transaction, they must unlock their payment app first. After unlocking is complete, the transaction proceeds as shown in Figure 5. The merchant sends a SELECT message to request the card to provide the type of card it supports. After the message is received, the card responds to the merchant with the application identifier (AID) of the phone-supporting card type, and the merchant then returns the selected AID. After the message is received, the phone generates a random number $R_{p}$ and sends the file control information (FCI) message required for subsequent messages, where FCI = (PDOL,$R_{p})$. Because the Processing Options Data Object List (PDOL) must be used in the next command to send the protocol’s new certificate data, EMV’s optional tag is used to indicate data the merchant must send in the PDOL field in the next command GET PROCESSING OPTIONS. In addition, we followed EMV’s Book 3 specifications [34] to create a new tag providing returned messages with a new field to send the random number $R_{p}$ to the merchant.

Next, Messages 3–4 are as illustrated in Figure 5. The protocol of mutual authentication between the merchant and credit card employs EMV’s original three-level certificate verification mechanism [34].

The merchant first receives the response message, comprising the successfully selected card AID, the random number *R_p_*, and the control message FCI of the PDOL, requesting that the merchant send data. The merchant sends the command GET PROCESSING OPTIONS to return the completed PDOL field required by the card and three tags newly defined for the EPMAR to enable the card to verify the merchant’s message: a random number *R_m_*, the merchant’s certificate $Cert_{m}^{acq}$, and the acquiring bank’s certificate $Cert_{acq}^{fca}$, as well as the list of authentication methods supported by the credit card in the mobile phone as stipulated in the EMV protocol and the data that must be accessed afterwards. After the phone receives the message, it uses the public key $PK_{fca}$ to verify the source of $Cert_{acq}^{fca}$ and takes the acquiring bank’s public key $PK_{acq}$ q to verify the source of $Cert_{m}^{acq}$. If the two certificates are verified, then the phone implements the following:

- It retrieves the merchant’s public key $PK_{m}$ from the merchant’s $Cert_{m}^{acq}$.
- It increases the application transaction counter (ATC) by 1.
- It generates a random number *S_p_* and uses it as the key to hash $S_{mas}=HMAC_{S_{p}}\left(R_{p},\;R_{m}\right)$ and *TK*=$HMAC_{S_{mas}}\left(R_{p},\;R_{m}\right)$, and it writes the encrypted secret $E_{PK_{m}}\left(S_{p}\right)$ and encrypted certificates $E_{TK}\left(Cert_{emv}^{iss},\;Cert_{iss}^{fca}\right)$ into the first designated address of the application file locator (AFL).
- It sends the address to the merchant and then uses the command READ RECORD to sequentially read the AFL, which is the address of all the card information including authentication and transaction procedures.
- It sends the application interchange profile (AIP), which is tagged to indicate support for mutual authentication.

If verification fails, the transaction is aborted.

Message 5

After the merchant receives the message indicating support for mutual authentication, it uses GET DATA to request the card’s ATC. Mutual authentication commences.

Message 6

When the merchant receives an ATC message from the phone, the merchant sends the command READ RECORD containing the first AFL address to the phone. When the phone receives this command, it follows the command and retrieves the $E_{PK_{m}}\left(S_{p}\right)$ and $E_{TK}\left(Cert_{emv}^{iss},\;Cert_{iss}^{fca}\right)$, which are calculated in advance when Message 3 is received, from the address indicated in the AFL, and then sends it directly to the merchant, thus enabling the merchant that owns the corresponding private key to decrypt the data to protect the certificate $Cert_{emv}^{iss}$ containing private information PAN.

Messages 7~8

When the merchant receives the encrypted certificate $E_{PK_{m}}\left(S_{p}\right)$, it retrieves *S_p_* by using its private key $SK_{m}$ to decrypt $E_{PK_{m}}\left(S_{p}\right)$. It employs the secret value *S_p_* and the key hash function to create $S_{mas}=HMAC_{S_{p}}\left(R_{p},\;R_{m}\right)$ and *TK*=$HMAC_{S_{mas}}\left(R_{p},\;R_{m}\right)$, and it then uses the session key *TK* to decrypt $E_{TK}\left(Cert_{emv}^{iss},\;Cert_{iss}^{fca}\right)$, retrieving the certificates $Cert_{emv}^{iss}\;\mathrm{and}\;Cert_{iss}^{fca}$. Next, it uses the public key $PK_{fca}$ to verify the source of the issuing bank’s $Cert_{iss}^{fca}$ and the user’s $Cert_{emv}^{iss}$. If both certificates are verified, the phone adopts the following steps:

- It retrieves the card’s public key $PK_{emv}$ from the card’s certificate $Cert_{emv}^{iss}.$ It uses $S_{mas}$ as a key to hash all previously received messages and the ATC-calculated message authentication code (MAC) $HMAC_{S_{mas}}$*(*$R_{p}$*||*$R_{m}$*||*$Cert_{m}^{acq}$*||*$Cert_{emv}^{iss}$*||*$S_{p}$*||ATC)*, using the private key $SK_{m}$ to encrypt and produce $auth_{m}$*=* $E_{SK_{m}}$*(*$HMAC_{S_{mas}}$*(*$R_{p}$*||*$R_{m}$*||*$Cert_{m}^{acq}$*||*$Cert_{emv}^{iss}$*||*$S_{p}$*||ATC))*, where the ATC is the current transaction number, which ensures that both parties are in the same session.
- In an EPMAR, the merchant uses the RFU field of the command VERIFY to send $auth_{m}$ for the phone to authenticate the merchant.

When the phone receives the command VERIFY with $auth_{m}$, it uses the merchant’s public key $PK_{m}$ to retrieve the MAC from $auth_{m}$ and uses the previously stored messages, $S_{mas}$, and key hash function to calculate the MAC and establish whether the received MAC equals the calculated MAC. If both MAC values are equal, the merchant is authenticated. Subsequently, the phone implements the following:

The phone uses $S_{mas}$ to hash all previously received messages and uses the ATC to calculate a MAC. It then uses the private key $SK_{emv}$ to encrypt the MAC into $auth_{p}$*=* $E_{SK_{emv}}$*(*$HMAC_{S_{mas}}$*(*$R_{p}$*||*$R_{m}$*||*$Cert_{m}^{acq}$*||*$Cert_{emv}^{iss}$ *||*$S_{p}$*||*$auth_{m}$*||ATC))* and saves $auth_{p}$ in the AFL location of the second data set.

- The phone hashes ambient factor data into the MAC, after which it employs the generated session key to encrypt the transaction data ${\mathrm{Data}}_{emv}$, the ambient factor message $AmbientData_{p}$, and the MAC *H(*$AmbientData_{p}$*)*, all of which are subsequently used, into $E_{TK}$*(*$Data_{emv}$*,* $AmbientData_{p}$*, H(*$AmbientData_{p}$*)),* and saves it in the location of the third dataset in AFL.
- The phone returns a message to indicate successful authentication.

Otherwise, the phone returns a message to indicate failed authentication and aborts the transaction.

Message 9

When the phone receives the merchant’s command READ RECORD containing the second AFL address, the phone sends the preproduced $auth_{p}$ to the merchant. After the merchant receives $auth_{p}$, it decrypts $auth_{p}$ with the public key $PK_{emv}$, and retrieves the MAC from it. The merchant then uses $S_{mas}$ as a key to hash the previously saved messages to calculate the authentication code and compare the retrieved MAC with the calculated code for credit card authentication. If both codes match, authentication is successful, and mutual authentication is completed. 

In Message 3, GET PROCESSING OPTIONS is used to send the merchant and acquiring bank’s certificates to the user; after the mutual authentication phase, the user and merchant have verified each other’s identity. In the next phase, the user advances into the ambient authentication phase.

Phase 3 Ambient Authentication Phase

**Figure 6:**
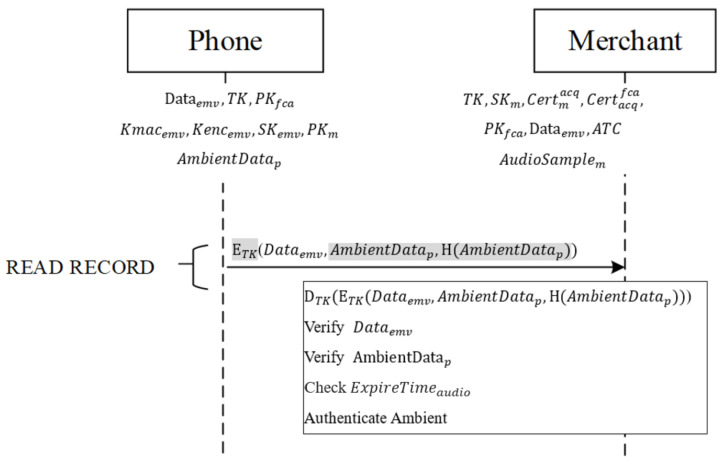
Ambient authentication phase.

After mutual authentication is completed, the mobile phone receives the merchant’s command READ RECORD containing the third AFL address, as shown in Figure 6, to request the data required for the transaction from the phone. After the phone receives this command, it sends the $E_{TK}$*(*$Data_{emv}$*,*$AmbientData_{p}$*,H(*$AmbientData_{p}$*))* produced in the authentication phase to the merchant.

After the message is received from the phone, the merchant decrypts it with the session key TK, retrieving $Data_{emv},AmbientData_{p}$*,H(*$AmbientData_{p}$*)*. First, the phone follows the EMV protocol, using the original static data authentication (SDA) and the issuing bank’s signed static data authentication (SSAD) in ${\mathrm{Data}}_{emv}$ to verify the integrity of other data ${\mathrm{Data}}_{emv}$. If the integrity is verified, the received $AmbientData_{p}$ is hashed, using the same hash function, into *H*${\left(AmbientData_{p}\right)}^{\prime }$*,* which is then compared with the hash value from the phone to determine whether they match. If the two match, the validity of $ExpireTime_{audio}$ from the phone is verified. If the valid time is valid, the verification result is written into b3 (the RFU field) of the terminal verification results’ (TVR’s) Byte 4. If all comparisons are correct and the ambient factor is received in time, then (1) to (3) are used to compare the received and self-recorded ambient factors [35]. Ambient factors are compared using different domains:

- Time domain: To test the similarity between time-based signals $X_{i}$ and $X_{j}$, their energy is first normalized such that the total energy of each signal equals 1. Next, a correlation or difference comparison is conducted. In the correlation comparison method, the correlation between every two signals is calculated, and the largest correlation is adopted:
  (1) $$S_{c}(i,j)=\max(\mathrm{Cross}\;-\;\mathrm{Corr}(X_{i},X_{j}))\;\mathrm{and}\;D_{c}\left(i,j)=1-S_{c}(i,j\right)$$

In the difference comparison method, the distance between the signal’s bit is calculated:

(2) $$D_{d}(i,j)=||X_{i}\;-\;X_{j}||\;\mathrm{and}\;S_{d}\left(i,j)=1-D_{d}(i,j\right)$$

- Frequency domain: Fast Fourier transform (FFT) is used to convert signals from the time domain to the frequency domain, and the frequency coefficient of each signal is calculated. Next, the correlation and the difference between the FFT coefficients are calculated to assess the similarity of the two.
- Time–frequency-based: Time domain and frequency domain results are combined, and (3) is employed:
  (3) $$D(i,j)=\sqrt{{\left(D_{c,time}\left(i,j\right)\right)}^{2}+{\left(D_{d,frequcncy}\left(i,j\right)\right)}^{2}}\;\mathrm{and}\;S\left(i,j)=1\;-\;D(i,j\right)$$

**Figure 7:**
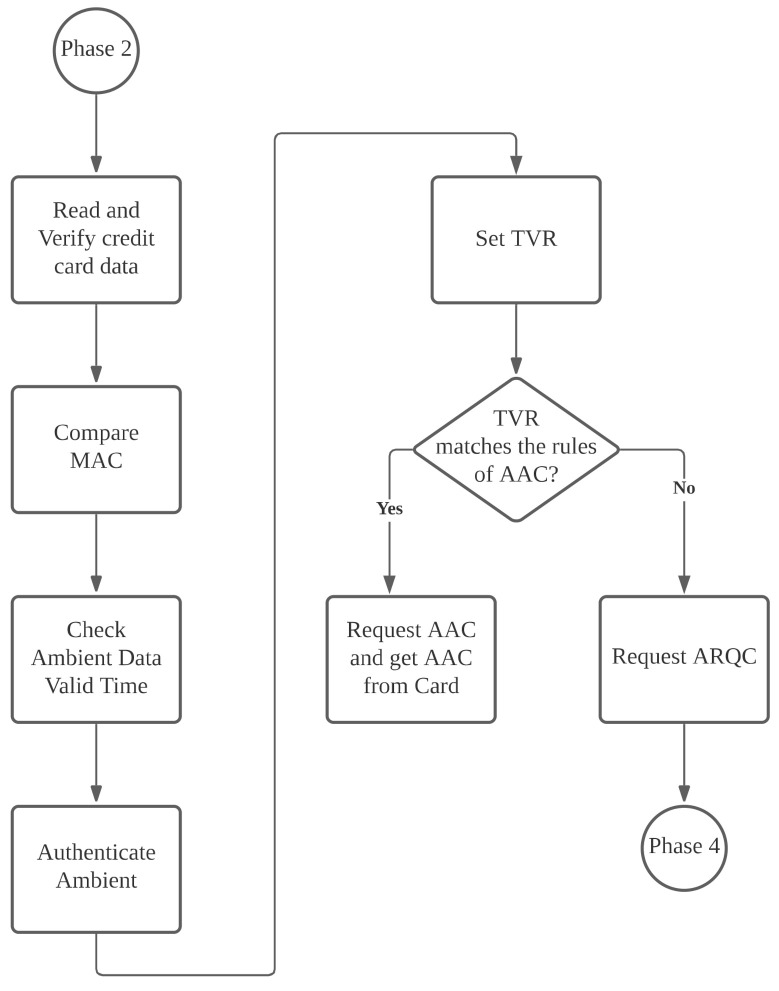
Ambient authentication phase.

If similarity is smaller than the threshold value, the verification result is written into b1 (RFU field) of TVR’s Byte 2. Finally, the merchant determines whether to reject the transaction or enter the transaction phase, depending on the TVR, as shown in Figure 7. 

**Figure 8:**
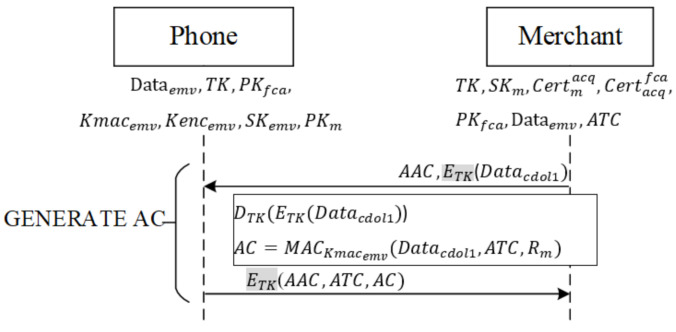
Rejection of transaction phase.

When the verification results do not match, and a merchant opts to reject a transaction, the merchant writes *CDOL1* in $Data_{emv}$ into $Data_{cdol1}$—the data required by the issuing banking requires for a transaction—and encrypts it with *TK*. As shown in Figure 8, the merchant follows EMV protocol specifications to transmit the command GENERATE AC containing AAC (rejection of transaction) and previously produced $Data_{cdol1}$ to the mobile phone. After the phone receives the command, it decrypts $E_{TK}\left(Data_{cdol1}\right)$ with *TK*, obtaining $Data_{cdol1}$.

Next, the phone follows EMV’s protocol content, employing the user–issuer shared key $Kmac_{emv}$ to hash the received $Data_{cdol1}$, the current transaction number ATC, and a random number $R_{m}$ obtained from the merchant during mutual authentication, into message *AC*. Finally, the phone encrypts *AAC*, *ATC*, and *AC* with *TK* and returns the encrypted message to the merchant.

If a user’s transaction is not rejected, the merchant requests that the phone generate an online Authorization Request Cryptogram (ARQC) and write *CDOL1* in $Data_{emv}$ into $Data_{cdol1}$, the data that the issuing bank requires for a transaction, to carry out the transaction.

Phase 4 Transaction Phase

**Figure 9:**
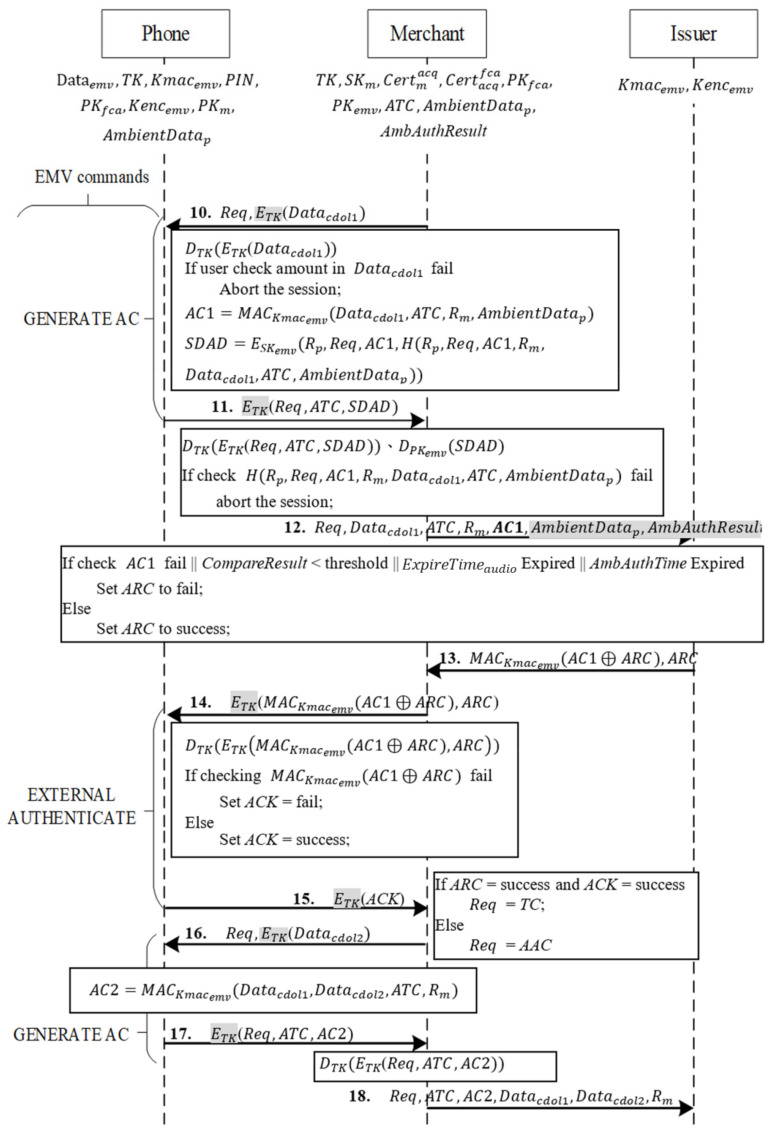
Transaction phase.

When the function in Phase 3 confirms a transaction, the transaction process is as shown in Figure 9. The merchant sends a request (*Req*) for an online transaction and uses the session key TK to encrypt $Data_{cdol1}$ from Phase 3 into $E_{TK}\left(Data_{cdol1}\right)$, and it then sends *Req* and $E_{TK}\left(Data_{cdol1}\right)$ to the phone with the command GENERATE AC.

Messages 10~11

When the phone receives the command GENERATE AC with *Req* from the merchant, it decrypts the transaction data with TK to obtain $Data_{cdol1}$. 

At this point, the transaction amount in $Data_{cdol1}$ is displayed on the phone screen for consumer verification. If its correct, the phone proceeds with three steps to generate the data required for EMV payment:

- It uses $Kmac_{emv}$, the key shared with the issuing bank, to hash the transaction data $Data_{cdol1}$ received from the merchant, the current transaction number *ATC*, and a random number $R_{m}$ from mutual authentication, as well as the ambient factor of the transaction $AmbientData_{p}$, into MAC *AC1*=$MAC_{Kmac_{emv}}\left(Data_{cdol1},\;ATC,\;R_{m},\;AmbientData_{p}\right)$.
- It hashes the random number from mutual authentication *R_p_*, the type of *AC*1 *Req*, *AC1*, and the data of *AC1*$\;Data_{cdol1},\;ATC,\;R_{m},AmbientData_{p}$.
- It uses the private key ${\mathrm{SK}}_{\mathrm{emv}}$ of the EMV credit card to generate SDAD from $R_{p},\;Req,\;AC1$, and the hash result $H(R_{p},Req,AC1,R_{m},Data_{cdol1},ATC,AmbientData_{p})$ from the preceding step.

Finally, the phone uses the session key TK to encrypt Req, ATC, and *SDAD* and transmits the encryption to the merchant.

Messages 12~13

After the merchant receives $E_{TK}\left(Req,ATC,SDAD\right)$ from the phone, it decrypts the received message with TK, obtaining Req, ATC, and *SDAD*. The merchant then uses the card’s public key $PK_{emv}$ to decrypt SDAD and verifies that its hashed value is correct. If the value is correct, the merchant sends $Req,\;Data_{cdol1},\;ATC,\;R_{m},\;AC1,\;AmbientData_{p}\;\mathrm{and}\;$*AmbAuthResult* to the issuing bank for online transaction. $AmbientData_{p}$ and *AmbAuthResult* are also sent for the issuing bank to verify whether the ambient factor has expired and whether it is within the valid period at the time of verification.

The bank uses shared key $Kmac_{emv}$, $\;Data_{cdol1}$ in the message, $ATC,\;R_{m},\;AmbientData_{p}$ to calculate the MAC value and compares it with the received *AC1* to determine whether they are equal. The bank then verifies whether the $ExpireTime_{audio}$ in $AmbientData_{p}$ has expired, whether *AmbAuthTime* in *AmbAuthResult* is within the $ExpireTime_{audio}$, and whether *CompareResult* matches the configured threshold value. 

If the message is correct, the issuing bank conducts a risk management check required for online transactions with the original credit card; for example, it checks whether the accumulated transaction exceeds the credit limit. If *AC1* is incorrect, the $ExpireTime_{audio}$ in $AmbientData_{p}$ has expired, *AmbAuthTime* in *AmbAuthResult* is not within the $ExpireTime_{audio}$, and *CompareResult* does not match the threshold value or the transaction is rejected after a risk management check, *ARC* is set as fail. Otherwise, *ARC* is set as success. Finally, the issuing bank follows EMV rules to use $Kmac_{emv}$ to produce the mutual exclusivity result of *ARC* and *AC* into $MAC_{Kmac_{emv}}\left(AC⊕ARC\right)$ with $Kmac_{emv}$ and sends *ARC* to the merchant.

Messages 14~15

After a merchant receives an authorization result from the bank, the merchant uses command EXTERNAL AUTHENTICATE to decrypt the bank’s message $MAC_{Kmac_{emv}}\left(AC⊕ARC\right)$ and ARC with TK and sends the decrypted message to the consumer’s phone. 

When the phone receives the message, it decrypts the message with session key TK, conducts XOR with the calculated AC and the decrypted ARC, uses the shared key $Kmac_{emv}$ to compute $MAC^{\prime }{}_{Kmac_{emv}}\left(AC⊕ARC\right)$, and checks whether the calculated MAC matches the received $MAC_{Kmac_{emv}}\left(AC⊕ARC\right)$. If both MAC match, ACK is set as success. Otherwise, ACK is set as fail. Finally, ACK is encrypted with TK and is returned to the merchant.

Messages 16~17

After the merchant receives a response from the phone, it unlocks the message with TK, obtaining ACK. If ARC in Message 13 is set as success and ACK is set as success, the merchant sets *Req* = TC to indicate that the transaction is complete. Otherwise, it sets *Req* = AAC to indicate that the transaction is declined. The merchant then uses TK to send $Data_{cdol2}$, the data required for the second GENERATE AC command as stipulated by the EMV protocol and the completed *Req* to the phone. When the user receives the second GENERATE AC command, the user follows the EMV protocol to hash all necessary data with the shared key $Kmac_{emv}$ to generate $AC2=MAC_{Kmac_{emv}}\left(Data_{cdol1},Data_{cdol2},ATC,\;R_{m}\right)$, and then uses TK to encrypt *Req, ATC*, and *AC2* to the merchant.

Message 18

After the merchant receives the message from the phone, it decrypts the message with TK and follows the original process to send the EMV-required data to the issuing bank for completion of the online transaction.

## 5. Experimental Results and Performance Analysis

### 5.1. Physical Environment

In our experiments, we used an NFC-enabled smartphone to simulate a consumer, and another smart phone to act as a POS. To simulate the real consumption environment, we performed experiments from areas where mobile payment is commonly used, including restaurants, convenience store A, convenience store B, and a hypermarket. 

The consumer’s phone featured a Qualcomm SDM845 2.8-GHz CPU, a 6-GB memory, and the Android 10 operating system. The phone used as a POS featured a Qualcomm S4 APQ8064 1.5-GHz CPU, a 2-GB memory, and the Android 5.1.1 operating system. We adopted the Android API Level 29 library to implement EMPAS’s and EMV’s consumer-end programs and the Android API Level 22 library to implement EMPAS’s and EMV’s merchant-end programs.

To analyze the lengths of protocol-required messages, we organized EMV-defined [34] data lengths, as shown in Table 2. For example, the SDAD used for card authentication is defined as Lr, where Lr denotes the length of the RSA key used. 

### 5.2. Ambient Factor Sampling and Comparison Method

In this experiment, when users pay with their mobile phones, the phone’s accelerometer value changes, and, when the phone is tapped on a POS, the POS’s accelerometer value also changes, providing an extremely intuitive method of triggering the POS for the subsequent action. Sound was used to represent the unique moment of every sampling. Therefore, we generated additional soundware of frequency 18 to 22 GHz to ensure the sampling of various ambient factors every time. We collected samples from the experiment areas and the sampling results were paired for comparison to ensure the ability of our protocol to detect two devices at the same location. Regarding audio comparison methods, Halevi et al. [35] found that time–frequency-based methods are the most accurate (53%), followed by frequency-based methods (50%), and time-based methods are the least accurate (38% and 14%). 

In our experiment, we selected the frequency-based method for comparison because, although the time–frequency-based method demonstrates the greatest accuracy, the correlation comparison of the frequency domain coefficient in equation (3) computed using our Sony Xperia XZ2 has exceeded EMV’s specification that the NFC between a phone and POS must be less than 100 ms. Frequency-based calculation using (1) takes 94 ms and, because NFC message-transmission time must be less than 100 ms, we adopted the frequency domain method in (1) to compare ambient sounds.

### 5.3. Comparison Results 

**Figure 10:**
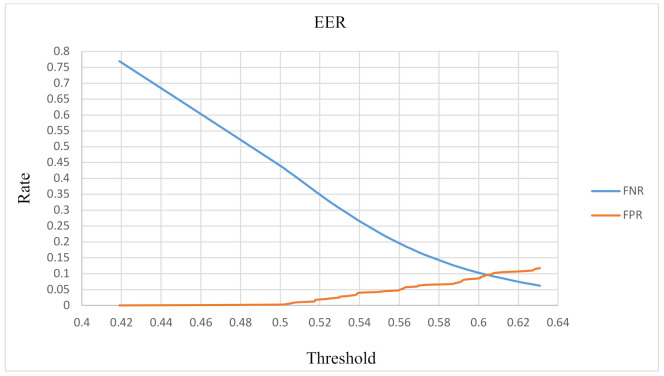
RoC curve.

Per the experimental results, various threshold values were used to calculate the false positive rate (FPR) and false negative rate (FNR). The FPR and FNR were calculated using (4), and the results were plotted onto a receiver operating characteristic (ROC) curve. As shown in Figure 10, when the threshold = 0.606, the point of intersection of the two curves is the equal error rate (9.6%) of this method. Our experimental results reveal that our use of an accelerometer combined with a comparison of ambient sounds exceeded 90% accuracy irrespective of whether an attacker was located in a space similar to the audio environment:

(4) $$\mathrm{FPR}=\frac{FP}{FP+TN},\;\mathrm{FNR}=\frac{FN}{FN+TP}$$

### 5.4. Execution Time

To ensure that the overall transaction time of our EMPAS protocol remains within the EMV-required transaction time, we analyzed our protocol’s total duration, including transmission time and computational time used by a POS and a mobile phone, to verify that the proposed verification procedure does not cause transaction failure.

According to the total message length required in Table 3, we used the fastest transmission speed of 6780 kbit/s when both NFC devices were in active mode for transmission to calculate our protocol-required transmission time, as shown in Table 4.

Our calculated computational time includes expressions for encryption or decryption, hash generation, random number generation, key generation, and ambient factor comparison, as indicated in Table 5 and Table 6. 

Because the EMV standards require a transaction to be completed in 500 ms [34], we defined protocol execution time as the sum of card computational time, merchant computational time, and message transmission time. As shown in Table 7, an RSA of 3072 bits causes the protocol to exceed the EMV-required time, but an RSA smaller than or equal to 2560 bits enables the protocol to complete a transaction within 500 ms. 

## 6. Security Analysis

Our protocol assumes that mobile phones have a secure storage and execution environment, that the merchant’s reader is secure, and that the reader communicates with the bank through a secure channel. In this section, we discuss how messages communicated between a phone and reader can be eavesdropped, tampered, and subjected to replay and relay attacks.

A. Mutual authentication:

Our method assumes that a mobile phone and a merchant’s reader can exchange and verify the certificates. Therefore, the phone and the reader can obtain each other’s public keys. Next, the reader uses its private key to encrypt all the received data into $auth_{m}$ and sends it to the phone in Message 7. The phone uses the merchant’s public key $PK_{m}$ to decrypt $auth_{m}$ and verify data. At this point, the phone has completed authenticating the merchant. Likewise, the phone sends the private-key-encrypted $auth_{p}$ in Message 9, and the merchant decrypts it and verifies the data, thus authenticating the phone.

B. Confidentiality:

In Message 3, the phone receives $R_{m}$ from the merchant, generates another secret value $S_{p}$, and creates a session key *TK*. In Message 6, the phone encrypts the secret value $S_{p}$ with the merchant’s public key. All messages after Message 6 are encrypted with this session key TK, thereby ensuring the confidentiality of the messages, and guaranteeing that transaction messages including the credit card number, transaction data, and the transaction amount are not accessed by a third party other than the merchant.

C. Prevention of replay attack:

Because messages following Message 6 are all encrypted by TK and because TK is created with the random numbers $R_{m}$, $R_{p}$, and $S_{p}$, an attacker cannot replay previous transaction messages, nor can they deceive a merchant because the current TK is different from the previous TK. Moreover, an ambient factor’s valid time is checked in the ambient authentication phase, and replaying a message causes a transaction to be declined because it has passed the valid time.

In each transaction, we created additional noise to ensure the sampling of various ambient factors every time to prevent replay attack. High-frequency sound decays as it is transmitted through the air. At more than one meter, the intruder will not be able to retrieve the original sample. When users pay with their mobile phones, the phone’s accelerometer value changes, and, when the phone is tapped on a POS, the POS’s accelerometer value also changes. The attacker cannot retrieve the accelerometer value. Therefore, it is difficult for an attacker to replay the message without being discovered by the user.

D. Integrity:

Messages in all transmitted data contain an MAC, such as Messages 3 and 6 or $auth_{p}$ and $auth_{m}$ in the MAC of Messages 7 and 9. In the ambient authentication phase, SSAD, the hash value of all data $Data_{emv}$ is retrieved from the card to confirm whether a message has been modified. In the transaction phase, message integrity is protected using the same method as that for existing credit card transactions. Therefore, if an attacker modifies a message, it can be detected by the MAC.

E. Non*-*repudiation*:*

With our protocol, a phone and merchant can authenticate each other, and the EMV’s transaction protocol in the phone requires a signed AC’s private key, $SK_{p}$, and a secure storage and computation environment. Our transaction process follows EMV standards, except that the merchant–phone communications are encrypted with a shared session key; therefore, our protocol and EMV protocol both possess nonrepudiation of card and merchant transactions.

F. Prevention of MITM attacks:

Because communications between a phone and merchant are encrypted, the phone and merchant perform mutual authentication to verify each other’s identity before ambient authentication and transaction are possible. In addition, attackers cannot launch replay attacks to pass mutual authentication and ambient authentication, and our protocol verifies message integrity. Attackers are discovered if they insert any message. Therefore, attackers cannot masquerade as a merchant or phone to launch MITM attacks.

G. Prevention of preplay attacks:

Bond et al. [14] indicated that a malicious merchant may record a transaction message and replay it in a legitimate store because the merchant’s random number generator is problematic. Our protocol provides mutual authentication, which requires attackers to communicate with merchants by using the correct private key before they can pass mutual authentication. Therefore, attackers cannot prerecord messages without the collaboration of a legitimate store. Because attackers cannot retrieve or create $auth_{m}$, which is exclusive to the merchant, they cannot generate the MAC $auth_{p}$ required for $auth_{m}$ in advance. During ambient factor sampling, attackers cannot easily make legitimate stores replay an audio frequency identical to that which has been prerecorded to pass the ambient authentication phase.

H. Prevention of relay attacks:

In the ambient factor sampling phase, we incorporated a challenge sound that is imperceptible to the human ear. The POS terminal and mobile phone simultaneously sampled the sound and assigned a valid time for the sample value. During the ambient authentication phase, the phone transmits the sampling result and valid time to the POS, and verification is performed by the POS. If similarity reaches the threshold value, the transaction commences; otherwise, the transaction is rejected. Similar ambient factors mean that both parties are located in a similar environment and, therefore, can be confirmed to be in close proximity, thereby preventing a relay attack. The summary of security analysis is shown in Table 8.

Next, we use the GNY logic to proof the correctness of our work [41]. The symbols used in the GNY logic proof can be found in Table 9.

**Table 9:** Notations of the proof.

P	Phone
M	Merchant
${\left\{X\right\}}_{K}$ $,\;{\left\{X\right\}}_{K}^{-1}$	Uses the symmetric key K to encrypt/decrypt the message X.
${\left\{X\right\}}_{+K}$ $,\;{\left\{X\right\}}_{-K}$	Uses the asymmetric key K to encrypt/decrypt the message X.
$H\left(X\right)$	Message X is protected by a one-way hash function $H\left(X\right)$.
P⊲X	P has received message X.
P∋X	P possesses message X.
P⊲∗X	P is told for X which he did not convey previously.
$P⫢♮\left(X\right)$	P believes X is fresh.
$P⫢⌀\left(X\right)$	P believes X is recognizable.
$P⫢P\overset{S}{↔}Q$	P believes S is shared by P and Q.


**A. Initial assumption:** In the beginning of the protocol, the phone holds ${\;\mathrm{Data}}_{emv},SK_{emv},Cert_{emv}^{iss},Cert_{iss}^{fca},PK_{fca}$ And the merchant holds So, $P∋Data_{emv}$ $P∋SK_{emv}$ $P∋PK_{fca}$ $P∋Cert_{emv}^{iss}$ $P∋{\left\{PK_{emv}\right\}}_{+SK}{}_{iss}$ $P∋Cert_{iss}^{fca}$ $P∋\;{\left\{PK_{iss}\right\}}_{+SK_{fca}}$ And $M∋SK_{m}$ $M∋PK_{fca}$ $M∋Cert_{m}^{acq}$ $M∋{\left\{PK_{m}\right\}}_{+SK_{acq}}$ $M∋Cert_{acq}^{fca}$ $M∋{\left\{PK_{acq}\right\}}_{+SK_{fca}}$ **B. Goal of the proof:** Goal #1: $M⫢P\overset{S_{p}}{↔}M$ (M believes he share $S_{p}$ with P) Goal #2: $P⫢M⫢P\overset{R_{p},\;R_{m}}{↔}M$ (Both M and P believe they share $R_{p},\;R_{m}$) Goal #3: $P⫢\;M⫢P\overset{TK}{↔}M$ (Both M and P believe they share TK) Goal #4: $P⫢\;P\overset{AmbientData_{p}}{↔}M$ (M believes he shares AmbientData*_p_*with P) **C. Proofple:** Mutual Authentication Phase: Message 2: $P∋R_{p}$ $M⊲\;*R_{p}$ $M⫢♮R_{p}$ $M⊲\;R_{p}$ (Rule T1) $M∋\;R_{p}$ (Rule P1) Message 3: $M∋\left(R_{m}\right)$ $P\;⊲\;*R_{m},\;*Cert_{m}^{acq},\;*Cert_{acq}^{fca}$ $P\;⫢♮*R_{m},\;*Cert_{m}^{acq},\;*Cert_{acq}^{fca}$ $P⊲\;R_{m},\;Cert_{m}^{acq},\;Cert_{acq}^{fca}$ (Rule T1) $P∋\;R_{m},\;Cert_{m}^{acq},\;Cert_{acq}^{fca}$ (Rule P1) $P\;∋R_{m},\;\;{\left\{PK_{m}\right\}}_{+SK_{acq}},\;{\left\{PK_{acq}\right\}}_{+SK_{fca}}$ $P\;∋PK_{acq}$ (Rule P8) $P\;∋PK_{m}$ (Rule P8) $P\;∋R_{m}\;$(Rule P1) $P\;∋PK_{acq}$ (Rule T1) $P\;∋PK_{m}$ (Rule T1) $P\;⫢♮R_{m}\;$(Rule F1) Message 4: $P⫢♮\left(S_{p}\right)$ Message 5: M⊲ ∗ AT M⫢♮ ATC P⊲ ATC (Rule T1) P∋ ATC (Rule P1) Message 6: $M\;⊲\;*{\left\{S_{p}\right\}}_{+PK_{m}},\;*{\left\{Cert_{emv}^{iss},Cert_{iss}^{fca}\right\}}_{TK}$ $M\;⫢♮\;*{\left\{S_{p}\right\}}_{+PK_{m}},\;*{\left\{Cert_{emv}^{iss},Cert_{iss}^{fca}\right\}}_{TK}$ $M\;⊲\;{\left\{S_{p}\right\}}_{+PK_{m}},\;{\left\{Cert_{emv}^{iss},Cert_{iss}^{fca}\right\}}_{TK}$(Rule T1) $M\;⊲\;S_{p}\;\;$(Rule T4) $M\;∋\;S_{p}$ (Rule P1) M ⫢$P\;∋\;S_{p}\;$(Rule I6) M ⫢ $P\overset{S_{p}}{\to }\;M$
**(Goal #1)** $M\;⊲\;\;Cert_{emv}^{iss},Cert_{iss}^{fca}$ (Rule T3) $M\;∋\;\;Cert_{emv}^{iss},Cert_{iss}^{fca}$ (Rule P1) Since $Cert_{emv}^{iss}={\left\{PK_{emv}\right\}}_{+SK}{}_{iss},\;Cert_{iss}^{fca}={\left\{PK_{iss}\right\}}_{+SK_{fca}},$ we got
$M\;∋\;{\left\{PK_{emv}\right\}}_{+SK}{}_{iss}$ $M\;∋{\left\{PK_{iss}\right\}}_{+SK_{fca}}$ $M\;∋\;PK_{iss}$ (Rule P8) $M\;∋\;PK_{emv}\;$(Rule P8) $M\;∋H_{S}{}_{p}\left(R_{p},\;R_{m}\right)\;$(Rule P2) $M\;∋\;S_{mas}$ $M\;∋\;H_{S_{mas}}\left(R_{p},\;R_{m}\right)\;$(Rule P2) M ∋TK $M\;∋E_{SK_{m}}(H_{mas}\left(R_{p}∥R_{m}∥Cert_{m}^{acq}∥\;Cert_{emv}^{iss}∥S_{p}∥ATC\right)$(Rule P2) $M∋E_{SK_{m}}(H_{mas}\left(R_{p}∥R_{m}∥Cert_{m}^{acq}∥\;Cert_{emv}^{iss}∥S_{p}∥ATC\right)$(Rule P2) Message 7: $P⊲\;*\;auth_{m}$ $P⫢♮\;auth_{m}$ $P⊲\;auth_{m}$ (Rule T1) $P∋\;auth_{m}$ (Rule P1) $P∋\;\{H_{S}{}_{mas}{\left(R_{p}∥R_{m}∥Cert_{m}^{acq}∥Cert_{emv}^{iss}∥S_{p}∥ATC\right\}}_{+SK_{m}}$ $P⊲H_{S}{}_{mas}\left(R_{p}∥R_{m}∥Cert_{m}^{acq}∥Cert_{emv}^{iss}∥S_{p}∥ATC\right)$ (Rule T6) $P⫢♮\;\left(R_{p}∥R_{m}∥Cert_{m}^{acq}∥Cert_{emv}^{iss}∥S_{p}∥ATC\right)$(Rule F11) $P⫢M\;∋\left(R_{p}∥R_{m}∥Cert_{m}^{acq}∥Cert_{emv}^{iss}∥S_{p}∥ATC\right)$ (Rule I6) $P⫢M\;∋\left(R_{p}∥R_{m}\right)$
**(Goal #2)** Message 9: $M\;⊲\;*auth_{p}$ $M⫢♮\;auth_{p}$ $M\;⊲auth_{p}$ (Rule T1) $M\;∋auth_{p}$ (Rule P1) $M∋\{H_{s}{}_{mas}{\left(R_{p}∥R_{m}∥Cert_{m}^{acq}∥Cert_{emv}^{iss}∥S_{p}∥auth_{m}∥ATC\right\}}_{SK_{emv}}\;$ $M∋H_{s}{}_{mas}(R_{p}∥R_{m}∥Cert_{m}^{acq}∥Cert_{emv}^{iss}∥S_{p}∥auth_{m}∥ATC)\;$(Rule T6) $M⫢♮\left(R_{p}∥R_{m}∥Cert_{m}^{acq}∥Cert_{emv}^{iss}∥S_{p}∥auth_{m}∥ATC\right)$ (Rule F11) $M⫢P\;∋\left(R_{p}∥R_{m}∥Cert_{m}^{acq}∥Cert_{emv}^{iss}∥S_{p}∥auth_{m}∥ATC\right)$ (Rule I6) $M⫢P\;∋\left(R_{p}∥R_{m}\right)$ $P⫢M⫢P\overset{R_{p},\;R_{m}}{↔}M$ (Rule J2), **(Goal #3)** Ambient Authentication Phase (Phone side) $P∋Data_{emv},\;$ $P∋AmbientData_{p}\;$ P∋TK $P∋H\left(AmbientDat\right)$ (Rule P4) $P∋{\left\{Data_{emv},\;AmbientData_{p},\;H\left(AmbientData_{p}\right)\right\}}_{TK}$ (Rule P6) (Merchant side) $M⊲\;*{\left\{Data_{emv},\;AmbientData_{p},\;H\left(AmbientData_{p}\right)\right\}}_{TK}$ $M⫢♮\;{\left\{Data_{emv},\;AmbientData_{p},\;H\left(AmbientData_{p}\right)\right\}}_{TK}$ M∋TK $M⊲\;{\left\{Data_{emv},\;AmbientData_{p},\;H\left(AmbientData_{p}\right)\right\}}_{TK}$ (Rule T1) $M∋\;{\left\{Data_{emv},\;AmbientData_{p},\;H\left(AmbientData_{p}\right)\right\}}_{TK}$. (Rule P1) $M⊲\;Data_{emv},\;AmbientData_{p},\;H\left(AmbientData_{p}\right)$
(Rule T3) $M⫢P\overset{Data_{emv},\;AmbientData_{p}}{↔}M$ (Rule J2), **(Goal #4)**


## 7. Conclusions

We proposed an EMV-compatible transaction protocol that not only incorporates three additional commands in the mutual authentication phase to complete mutual authentication, but also involves the same transaction-required commands and sequences used in EMV protocols.

In our protocol, ambient authentication is used to prevent relay attacks. Through the addition of a challenge sound to the original ambient sound, we prevented attackers from passing ambient authentication and completing a transaction in a similar environment.

Compared with the original EMV protocol, our protocol authenticates merchants, encrypts sent messages, and achieves ambient authentication. Our protocol has the ability to detect whether the consumer and the POS are at the same location.

Our approach is not limited to the use of sound as ambient authentication. We use the phone’s accelerometer as another authentication factor. The attacker cannot retrieve the accelerometer value. Therefore, it is difficult for an attacker to replay the message without being discovered by the user.

In addition, the proposed protocol prevents the majority of known attacks in the original EMV protocols, such as MITM and clone attacks, and concurrently protects personal credit card information. Although mutual authentication, ambient authentication, and message encryption considerably increase the computation, message transmission, and storage loads of our protocol compared with EMV protocols, our experimental analysis results reveal that, for an RSA smaller than 2560 bits, the proposed protocol enables a transaction to be performed within EMV’s time requirement of 500 ms.

## Figures and Tables

**Table 1:** Notations.

$Cert_{target}^{publisher}$	A certificate issued by a publisher to a target. For example, $\;{Cert}_{iss}^{fca}$ is issued by a financial certificate authority (FCA) to an issuing bank (issuer). The certificate contains the public key corresponding to the target’s private key
$PK_{target}$	A target’s public key, for example, $PK_{iss}$ is an issuing bank’s public key
$SK_{target}$	A target’s private key, for example, $SK_{iss}$ is an issuing bank’s private key
$Kenc_{emv}$	Symmetric keys shared between a credit card and issuing bank and are used for encryption
$Kmac_{emv}$	Symmetric keys shared between a credit card and issuing bank and used to calculate a message authentication code
$R_{m}$, $R_{p}$	Random numbers
$S_{p}$	A secret value randomly generated by the phone
$S_{mas}$	A secret value used to generate the session key *TK*
TK	A session key used by a phone to communicate with a store
$E_{key}\left(M\right)$	Encryption of message *M* with a symmetric or asymmetric key, where *key* is the key used; for example, $E_{Kenc_{emv}}\left(M\right)$ is the encryption of message *M* with $Kenc_{emv}$
$D_{key}\left(M\right)$	Decryption of message *M* with a symmetric or asymmetric key, where *key* is the key used; for example, $D_{Kenc_{emv}}\left(M\right)$ is the decryption of message *M* with $Kenc_{emv}$
$MAC_{Kmac_{emv}}\left(M\right)$	A function in the EMV protocol using the symmetric encryption function and key $Kmac_{emv}$ to calculate transaction message *M*’s message authentication code
$HMAC_{key}\left(M\right)$	A function using *key* to hash message 𝑀 into a message authentication code
$H\left(M\right)$	Hash of message *M* as a message authentication code
$AudioSample_{target}$	A target’s audio-sampled ambient data; for example,$\;AudioSample_{p}$ is the sampled ambient data of a phone
$ExpireTime_{target}$	A target’s valid time, for example, $ExpireTime_{audio}$ is the audio’s valid time
$AmbientData_{target}$	A target’s sampled ambient data, which contains the sample data and sample data’s valid time; for example,$\;AmbientData_{p}$ is the ambient data sampled from a phone
*CompareResult*	Sampled ambient data comparison results
*AmbAuthTime*	System time to complete ambient authentication
*AmbAuthResult*	Results of ambient authentication. This information contains ambient data comparison results and the system time to complete ambient authentication

**Table 2:** EMV data lengths.

EMV’s Existing Data	Length (Bytes)
$Data_{emv}$	*L_r_* + 67
ATC	2
Card and merchant certificate $(Cert_{emv}^{iss},\;Cert_{m}^{acq}$)	*L_r_* + 42
Bank’s certificate $(Cert_{iss}^{fca}$$,Cert_{acq}^{fca}$)	*L_r_* + 36
*Type*	20
$R_{p},\;R_{m}$	4
*Req, TC*, *ARQC*, *AAC*, *OCRC*	1
SDAD	*L_r_*
*ACK*	1
*AIP+AFL*	38
*ARC*	2
$Data_{cdol1}$	45
$Data_{cdol2}$	8
AC1, AC2	8
New Data	Length (bytes)
$Cert_{emv\_off}^{iss}$	*L_r_* + 42
$auth_{p},\;auth_{m}$	*L_r_*
$S_{p},\;S_{mas}$	48
*PDOL*	34
$AmbientData_{p}$	89 K
AmbAuthResult	1

**Table 3:** Online message length (bytes).

	RSA 1024 bits	RSA 1536 bits	RSA 2048 bits	RSA 2560 bits	RSA 3072 bits
EMPAS-AES 128	90,822	91,462	92,134	92,742	93,388
EMPAS-AES 192	90,870	91,526	92,134	92,790	93,452
EMPAS-AES 256	90,934	91,574	92,214	92,854	93,500
EMPAS-Cert-AES 128	90,949	91,653	92,357	93,061	93,771
EMPAS-Cert-AES 192	90,973	91,693	92,365	93,085	93,811
EMPAS-Cert-AES 256	91,013	91,717	92,421	93,125	93,835

**Table 4:** Comparison of message transmission time (ms).

	RSA 1024 bits	RSA 1536 bits	RSA 2048 bits	RSA 2560 bits	RSA 3072 bits
EMPAS-AES 128	107.16	107.92	108.71	109.43	110.19
EMPAS-AES 192	107.22	108.00	108.71	109.49	110.27
EMPAS-AES 256	107.30	108.05	108.81	109.56	110.32
EMPAS-Cert-AES 128	107.31	108.15	108.98	109.81	110.64
EMPAS-Cert -AES 192	107.34	108.19	108.99	109.83	110.69
EMPAS-Cert -AES 256	107.39	108.22	109.05	109.88	110.72

**Table 5:** Online consumer-end phone computational time (ms).

	RSA 1024 bits	RSA 1536 bits	RSA 2048 bits	RSA 2560 bits	RSA 3072 bits
EMPAS-AES 128	11.58	29.12	61.38	110.9	182.22
EMPAS-AES 192	12.18	29.72	61.98	111.5	182.82
EMPAS-AES 256	12.3	29.84	62.1	111.62	182.94
EMPAS-Cert-AES 128	11.73	29.54	62.14	112.13	183.95
EMPAS-Cert -AES 192	12.18	29.99	62.59	112.58	184.4
EMPAS-Cert -AES 256	12.27	30.08	62.68	112.67	184.49

**Table 6:** Online merchant–end reader computational time (ms).

	RSA 1024 bits	RSA 1536 bits	RSA 2048 bits	RSA 2560 bits	RSA 3072 bits
EMPAS-AES 128	107.24	125.76	159.58	206.8	281.63
EMPAS-AES 192	107.84	126.36	160.18	207.4	282.23
EMPAS-AES 256	107.96	126.48	160.3	207.52	282.35
EMPAS-Cert-AES 128	106.92	125.44	159.26	206.48	281.31
EMPAS-Cert -AES 192	107.37	125.89	159.71	206.93	281.76
EMPAS-Cert -AES 256	107.46	125.98	159.8	207.02	281.85

**Table 7:** Protocol execution time (ms).

	RSA 1024 bits	RSA 1536 bits	RSA 2048 bits	RSA 2560 bits	RSA 3072 bits
EMPAS-AES 128	225.98	262.8	329.67	427.13	574.04
EMPAS-AES 192	227.24	264.08	330.87	428.39	575.32
EMPAS-AES 256	227.56	264.37	331.21	428.7	575.61
EMPAS-Cert-AES 128	225.96	263.13	330.38	428.42	575.9
EMPAS-Cert -AES 192	226.89	264.07	331.29	429.34	576.85
EMPAS-Cert -AES 256	227.12	264.28	331.53	429.57	577.06

**Table 8:** Protocol security analysis.

	EMPAS	EPMAR [17]	Original EMV (with CDA)	Original EMV (with DDA)	Original EMV (with SDA)
Mutual authentication	◯	◯	△ ^1^	△ ^1^	△ ^1^
Confidentiality	◯	◯	✕	✕	✕
Prevention of replay attack	◯	◯	◯	◯	◯
Data privacy	◯	◯	✕	✕	✕
Integrity	◯	◯	◯	◯	◯
Nonrepudiation	◯	◯	◯	△ ^2^	△ ^2^
Prevention of MITM attack	◯	◯	✕	✕	✕
Prevention of preplay attacks	◯	◯	✕	✕	✕
Prevention of relay attacks	◯	△ ^3^	△ ^4^	△ ^4^	△ ^4^

◯: Indicates that the attack can be prevented. ✕: Indicates that the attack cannot be prevented. △ ^1^: EMV authenticates only the card and not the POS. △ ^2^: DDA and SDA can achieve only a merchant’s nonrepudiation and not a consumer’s nonrepudiation. △ ^3^: EPMAR relies only on the payment amount displayed on the phone screen for consumers to verify whether the purchase amount matches, but if a consumer’s phone screen can be controlled by an attacker, such prevention methods are ineffective. △ ^4^: EMV proposes distance-bounding for prevention, but this method requires a precise counter and is therefore unsuitable for commercial phones.

## Data Availability

Not applicable.
